# Chemical Composition, Antioxidant, Anti-Bacterial, and Anti-Cancer Activities of Essential Oils Extracted from *Citrus limetta* Risso Peel Waste Remains after Commercial Use

**DOI:** 10.3390/molecules27238329

**Published:** 2022-11-29

**Authors:** Arunaksharan Narayanankutty, Naduvilthara U. Visakh, Anju Sasidharan, Berin Pathrose, Opeyemi Joshua Olatunji, Abdullah Al-Ansari, Ahmed Alfarhan, Varsha Ramesh

**Affiliations:** 1 Division of Cell and Molecular Biology, PG & Research Department of Zoology, St. Joseph’s College (Autonomous), Calicut 673008, India; 2 Department of Agricultural Entomology, College of Agriculture, Kerala Agricultural University, Thrissur 680656, India; 3 African Genome Center, Mohammed VI Polytechnic University, Ben Guerir 43150, Morocco; 4 Traditional Thai Medical Research and Innovation Center, Faculty of Traditional Thai Medicine, Prince of Songkla University, Hat Yai 90110, Thailand; 5 Department of Botany and Microbiology, College of Science, King Saud University, P.O. Box 2455, Riyadh 11451, Saudi Arabia; 6 Department of Biotechnology, Deakin University, Geelong, VIC 3217, Australia

**Keywords:** *Citrus limetta*, antioxidant activity, essential oil, antibacterial activity, anticancer activity

## Abstract

Citrus plants are widely utilized for edible purposes and medicinal utility throughout the world. However, because of the higher abundance of the antimicrobial compound D-Limonene, the peel waste cannot be disposed of by biogas production. Therefore, after the extraction of D-Limonene from the peel wastes, it can be easily disposed of. The D-Limonene rich essential oil from the *Citrus limetta* risso (CLEO) was extracted and evaluated its radical quenching, bactericidal, and cytotoxic properties. The radical quenching properties were DPPH radical scavenging (11.35 ± 0.51 µg/mL) and ABTS scavenging (10.36 ± 0.55 µg/mL). There, we observed a dose-dependent antibacterial potential for the essential oil against pathogenic bacteria. Apart from that, the essential oil also inhibited the biofilm-forming properties of *E. coli*, *P. aeruginosa*, *S. enterica*, and *S. aureus*. Further, cytotoxicity was also exhibited against estrogen receptor-positive (MCF7) cells (IC_50_: 47.31 ± 3.11 µg/mL) and a triple-negative (MDA-MB-237) cell (IC_50_: 55.11 ± 4.62 µg/mL). Upon evaluation of the mechanism of action, the toxicity was mediated through an increased level of reactive radicals of oxygen and the subsequent release of cytochrome C, indicative of mitotoxicity. Hence, the D-Limonene rich essential oil of *C. limetta* is useful as a strong antibacterial and cytotoxic agent; the antioxidant properties exhibited also increase its utility value.

## 1. Introduction

Citrus fruits are widely consumed and, as a result, the waste products in the form of peels are accumulating [1]. Sustainable waste management is highly dependent on the conversion of waste materials into biogas or other forms of energy [2]. However, because of the presence of the antibacterial compound D-Limonene, the peels of citrus fruits are less utilizable in biogas production. Studies have indicated that the D-Limonene present in citrus peels inhibits the anaerobic digestion of the waste by preventing the growth of bacterial colonies [3,4]. In addition, studies have also indicated that the extraction of the D-Limonene and other bactericidal compounds from the citrus peel enhances the anaerobic digestion and subsequent conversion of these waste products into biogas [5,6]. Hence, the extraction of D-limonene from the citrus peel is of high significance and, also, the compound has commercial and pharmacological uses [7,8].

Among the major citrus species, *Citrus maxima, Citrus limetta, Citrus limon, Citrus aurantifolia*, and *Citrus reticulata* are widely used and consumed in world markets [9]. The major method of extraction of bioactive D-Limonene from peel waste is in the form of essential oils [10,11,12]. The essential oil extracted from the *Citrus reticulata,* the commonly found variety among citrus fruits, was found to inhibit the growth of microbes [13,14], as well fungal strains such as *Penicillium italicum* and *P. digitatum* [15]. The radical quenching and bactericidal potentials of the essential oil is also reported that subsequently resulted in its wound-healing properties [16]. Similarly, antibacterial activities are also reported for essential oil isolated from *C. maxima* by different extraction methods [17]; likewise, this essential oil was also effective against pests of stored products [18]. The growth inhibitory potential against *Aspergillus flavus,* the producer of the common food toxicant aflatoxin, was also observed [19]. The *Citrus limetta* essential oil exhibited strong larvicidal activity against the *Anopheles* and *Aedes* mosquitoes [20]; the essential oil was also effective against skin diseases with underlining inflammation or oxidative stress in cell culture models and animals [21]. The essential oil of *Citrus medica L. var. sarcodactylis* was found to be a good antibacterial agent by altering the membrane integrity of the different microbial strains [22]. Anticancer activities are also attributed to citrus-derived essential oils. A study by Yang, et al. [23] indicated that the gannan navel orange (*C. sinensis*) exerts antiproliferative potential against human prostate and lung cancer cells. *Citrus medica* is another plant that is shown to have anti-neoplastic activities against colorectal cancer cells [24]. The *C. limon* essential oil-nanoemulsion has been found to exert apoptosis in A549 cells under in vitro conditions [25]. A study by Elansary, et al. [26] compared the anticancer potential of *C. aurantifolia, C. limon,* and *C. paradisi* against various cancer cell lines. Among these, *C. paradisi*, was most effective and capable of inducing apoptosis.

The utility of various essential oils extracted from peels, fruits, and leaves of different citrus plants is available. However, the novelty of the work is that the source of essential oil used for the analysis was the peel waste of commercially used *Citrus limetta,* and it was collected from juice shops. Further, we analyzed the constituent compounds and potentials of the essential oil as an antioxidant, bactericidal, and cytotoxic agent.

## 2. Results

### 2.1. The Average Yield and Volatile Content in the Peel Essential Oil of Citrus limetta

**Figure 1:**
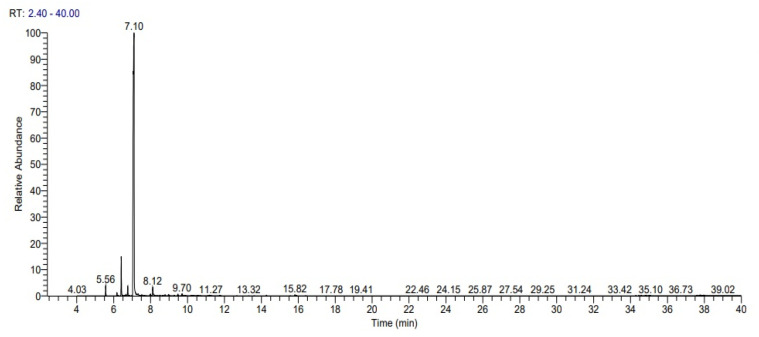
The total ion chromatogram of the peel of the *C. limetta* essential oil.

The average yield of essential oils from the waste peels of *C. limetta* was 0.63%. The GC–MS chromatogram of essential oil isolated from the peels of *Citrus limetta* (Figure 1) shown the occurrence of D-limonene and α-myrcene as chief components (Table 1).

### 2.2. Anti-Radical Activities

The anti-radical abilities of the peel of the CLEO indicated a higher activity compared to the major compound S-limonene. On contrary, the ascorbic acid (standard compound) was more active in scavenging DPPH free radicals and ferric-reducing properties. However, the lipid peroxidation inhibition and hydrogen peroxide scavenging were high in the CLEO (*p* < 0.05). The ABTS radical scavenging potential was found to be similar in both CLEO and ascorbic acid (Table 2).

### 2.3. Cytotoxicity of the C. limetta Essential Oil, D-Limonene, and Cyclophosphamide

**Figure 2:**
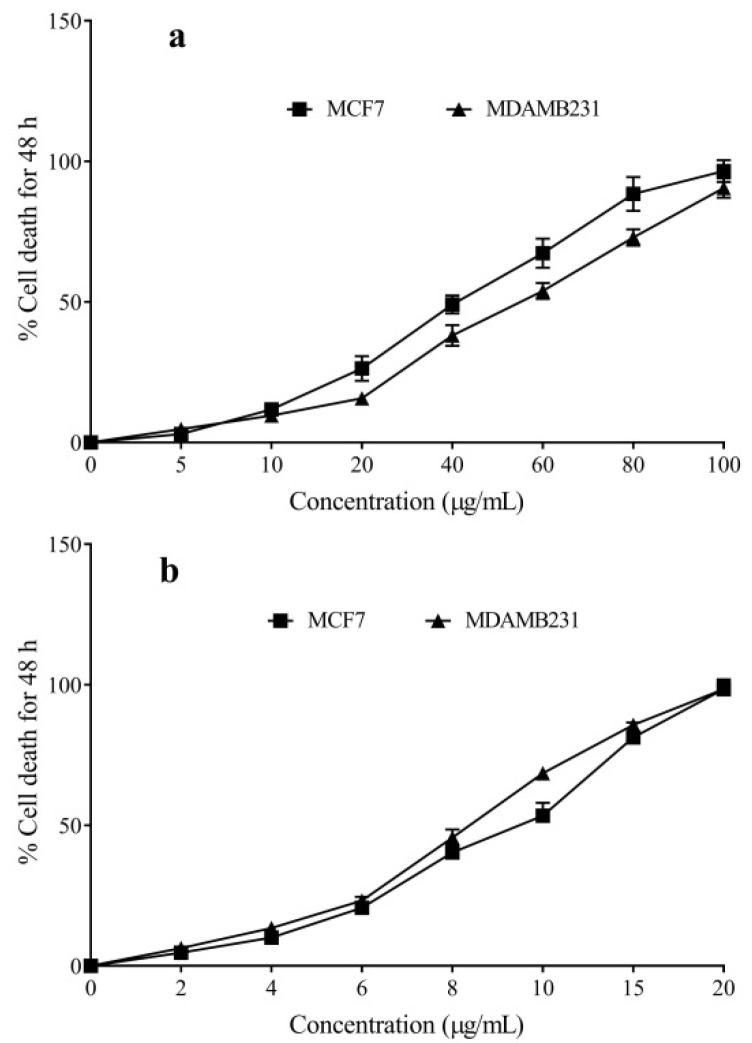
Anticancer activity of the *Citrus limetta* essential oil (**a**) and cyclophosphamide (**b**) was analyzed against MCF7 and MDAMB-231 cells.

The essential oil treatment-induced dose-dependent cytotoxicity against MCF7 and MDAMB-231 cell lines (Figure 2).

The cytotoxicity of the CLEO, D-Limonene and cyclophosphamide were indicated as the IC_50_ values in Table 3. The anticancer activity of the CLEO was high against MCF7 cells; whereas, in the MDAMB231 cells, the IC_50_ value was high. However, the standard drug, cyclophosphamide, had significantly higher activity compared to the CLEO (Table 3). However, the cytotoxicity of D-Limonene was low compared to the essential oil. The morphological changes are indicated in the Figures S1 and S2 (Supplementary Materials).

**Figure 3:**
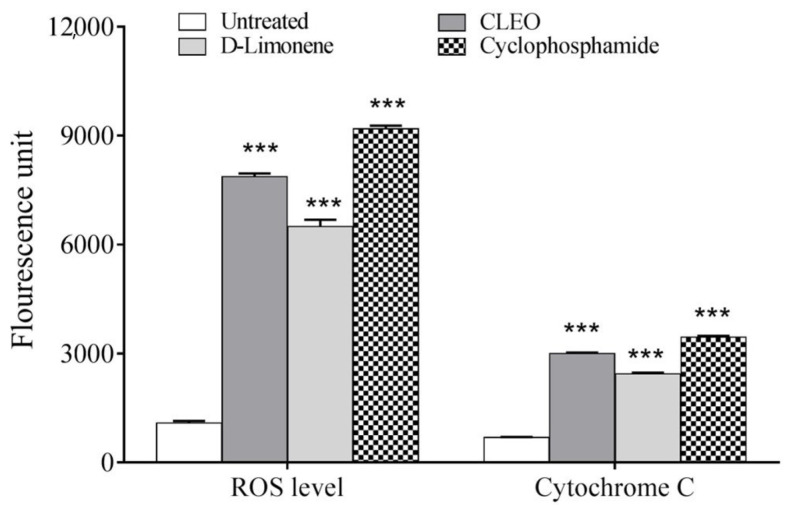
The mechanistic bases of action of the Citrus limetta peel essential oil along with the standard anticancer drug, cyclophosphamide, at their respective IC_50_ values. (*** indicate *p* < 0.001).

The mechanism of action was estimated in terms of the reactive oxygen species generated in the cells and also based on the release of mitochondrial cytochrome C release. There observed a noteworthy elevation in the ROS levels of cells treated with the IC_50_ value equivalent dose of different compounds (Figure 3) and a subsequent increase in the release of cytochrome C levels.

### 2.4. Bactericidal Properties of the CLEO

The bactericidal activity of the CLEO was indicated in Table 4. The disc diffusion assay observed significant inhibition of bacterial growth in the *Citrus limetta* essential oil treatment. The highest activity was observed against *Staphylococcus aureus* (Table 4). The CLEO showed moderate activities against other bacterial strains.

Further, the MIC value of the CLEO was estimated against the same microbial species (Table 5). The CLEO had the lowest level of MIC value against *E. coli* (0.50 ± 0.03 mg/mL) and *S. aureus* (0.50 ± 0.02 mg/mL). The standard antibiotic gentamicin had been more effective, as shown in Table 5.

The anti-biofilm formation activity of CLEO and gentamicin was also determined. As indicated in Table 6, we observed significant anti-biofilm formation (0.5 mg/mL) for CLEO compared to that of the standard antibiotic gentamicin.

## 3. Discussion

Various products from Citrus plants are widely consumed fruits and source for various nutrients and pharmacologically active agents. However, fruits also contribute to large quantities of waste products, as in other agriculture sectors [27,28]. The predominant waste products from various citrus plants include their peel wastes; these waste products later decay and lead to pollution at various levels [2]. However, these waste peels also emerge as important sources of biological and pharmacologically active essential oils [5,6]. Hence, the present study analyzed the potentials of citrus peel-derived essential oils as anticancer and antibacterial agents using in vitro experimental models.

The gas chromatography analysis indicated the presence of *D-limonene* as major component in CLEO. The predominant compound in the essential oil was *D-limonene*. The highest level was observed in *Citrus limetta, Citrus reticulata,* and *Citrus limon.* Limonene is an important bioactive compound that is shown to have strong antibacterial and antifungal properties, and thereby acts as a potent agent against microbial diseases [29,30,31]. The D-Limonene is also reported to have significant anticancer potentials; the mechanistic basis of action is proven to be mediated by autophagy and apoptosis in various cancer cells [32,33,34]. The compounds α-pinene and α-myrcene are are the other minor constituents present in the essential oil; they are also shown to have potent antimicrobial, anti-inflammatory, and antitumor properties [35,36]. The presence of these compounds at a lower level is noted in various citrus essential oils prepared from leaves or fruits [37,38,39,40,41].

Furthermore, the bactericidal properties of the *C. limetta* essential oil and its bioactive compound D-Limonene are also observed. The bacterial strains tested are pathogenic to animals and humans [42,43]. Previous studies using different citrus essential oils also indicated the antibacterial potentials [41,44,45,46]. A previous study has indicated that the inhibition zone of *Citrus* spp. was in the range of 5.8–21.0 mm for *E. coli* and the same was 5.0–10.0 mm for *Lactbacillus plantarum* [47]. A study has also observed that the inhibition zones were in the range of 14.0–26.0 mm for various bacteria, and the minimum inhibitory concentration value was varied between 0.039 and 2.5 mg/mL [48]. The bactericidal properties are ascribed to *D*-limonene, present in essential oils [29,30,31]. D-Limonene in the peels is also known to cause issues in the anaerobic degradation during biogas [3,4].

The CLEO induced anticancer effects in MCF7 and MDAMB231 cells. Previous reports have indicated that the citrus essential oil IC_50_ values against human lung cancer cells are estimated to be 17.53–45.74 µg/mL [23,49]. The MCF7 cells are considered to be estrogen receptor-positive cells and MDAMB231 is a triple-negative breast cancer cell [50,51]. Hence, it can be possible that essential oils exert anticancer properties in both types of breast cancer cells. This will open up a new source of anticancer agents against different types of breast cancers. The bioactive compounds present in the *Citrus limetta* essential oil, such as limonene [32,52], citral [53,54], and terpineol [55,56], are strong anti-proliferative and apoptotic agents in cancer cells. Hence, the bioactive compounds present in the CLEO might be accountable for these activities.

## 4. Materials and Methods

### 4.1. Essential Oil Extraction from the Peel Waste of Citrus limetta

Peels of *Citrus limetta* were obtained from juice shops in Kerala, India (10.5276° N, 76.2144° E). After washing, the peels were extracted by hydro-distillation in a Clevenger-type apparatus for 4–5 h (100 °C). The final yield from the peels (CLEO) was represented as mg of CLEO obtained per gram of fresh peels (%, *V/w*). The dehydration of CLEO was performed using sodium sulfate (AR) and kept in amber-colored bottles in refrigerated conditions.

### 4.2. Analysis of the Component Chemicals in CLEO

The chemical constituents are analyzed using a TSQ 8000 Evo GC-MS system (Thermoscientific, Waltham, MA, USA) furnished with an autosampling system and TG-5MS column as described by our previously published method [18]. The individual constituents were derived by matching the MS spectra of the NIST library. Further, a blank run was performed following each sample analysis to avoid contamination. The retention index (Kovats index) of individual compounds was calculated by the co-injection of the n-alkene mixture (C_7_-C_30_) passed through the column with maintaining the same conditions followed by essential oil chemical characterization. The calculated RI of each constituent was compared with their library RI.

### 4.3. Quenching Abilities of Citrus limetta Peel Essential Oil against Various Free Radicals

The radical quenching abilities were estimated using different models. Initially, different concentrations of the *Citrus limetta* essential oil were prepared in Tween 80 (0–100 µg/mL), likewise, the D-Limonene and ascorbic acid was also dissolved in dimethyl sulfoxide. The DPPH radical scavenging was estimated according to the procedures prescribed by House, et al. [57]. ABTS- quenching was performed by the methods mentioned by Baliyan, et al. [58]. The quenching of peroxide radicals was carried out using H_2_O_2_ as mentioned by Al-Amiery, et al. [59].

### 4.4. Anti-Proliferative Effect of the Citrus limetta Peel Essential Oil

The estrogen receptor-positive human breast cancer cell (MCF7) and human triple-negative breast cancer cell (MDAMB231) was received from the NCCS cell repository (Pune, Maharashtra, India) and cultured in complete DMEM media. The cytotoxicity analysis was carried out using an MTT assay as described earlier [60]. The cell death was measured spectrophotometrically at 570 nm and expressed as percentage using the following formula (Equation (1)):

(1) $$\%\;\mathrm{Cell}\;\mathrm{death}=\frac{\mathrm{OD}\;\mathrm{of}\;\mathrm{Control}-\mathrm{OD}\;\mathrm{of}\;\mathrm{Sample}}{\mathrm{OD}\;\mathrm{of}\;\mathrm{Control}}\times 100$$

### 4.5. Effect of the CLEO on ROS Level and Cytochrome C Release

The mechanism of action was determined in terms of the cytochrome-C release and reactive oxygen species levels; these changes in essential oil-treated cells were determined by commercially available kits, as described in our previous article [61]. The cells were treated with the respective IC_50_ value doses of different citrus peel essential oils for the mechanistic basis of action.

### 4.6. Analysis of Antibacterial Activity

#### 4.6.1. Bacterial Maintenance

The bacterial colonies of *Pseudomonas aeruginosa, Escherichia coli, Staphylococcus aureus,* and *Salmonella enterica* were procured from MTCC, Chandigarh, India. The bacteria were initially grown under standard atmospheric conditions; the procedures strictly adhered to the methods described in a previous study [62].

#### 4.6.2. Inhibition Zone Formation by *C. limetta* Essential Oil Treatment

The aforementioned bacteria were cultured using LB broth. For the disc diffusion assay, the MHA agar plate was inoculated with individual bacterial strains. Further, the *C. limetta* essential oil (10 μL), D-Limonene, and gentamicin were applied to Whatman No.1 filter paper (8 mm diameter) and placed in the MHA agar plates. The inhibition zones for each were determined after 24 h [63].

#### 4.6.3. *C. limetta* Essential Oil Minimum Inhibitory Concentrations (MIC)

The MIC value was estimated by the methods described by the standard methods described previously [64,65,66]. The different bacteria were set to 5 × 10^5^ CFU/mL density using a spectrophotometer. From this, about 50 µL was transferred to the individual wells of a 96-well plate together with the *Citrus limetta* essential oil, gentamicin, and D-Limonene. The media was then supplemented with 2,3,5-triphenyl tetrazolium chloride (TTC) (10 µL). The MIC concentration was determined to be the lowest concentration without pink color.

#### 4.6.4. Analysis of Biofilm Formation Inhibition by the *Citrus limetta* Essential Oil

The inhibition of biofilm formation by the *Citrus limetta* essential oil was carried out by the methods of Selim, et al. [67]. Briefly, the assay was carried out using a 96-well plate containing growing cells that were incubated with 5% concentrations of the *Citrus limetta* essential oil; the cells after 24h were stained with crystal violet.

### 4.7. Statistical Analysis

The final values of radical quenching assay, cytotoxicity analysis, and bactericidal studies were shown as mean ± standard deviation. These assays were repeated three times and each was performed in triplicate.

## 5. Conclusions

The agro-waste products of *Citrus limetta* plants are their peels; the results indicated that the peels are important sources of aromatic essential oils and, also, the predominant compound *D*-limonene, α-pinene, and α-myrcene. Results also indicated the radical quenching potential of the *C. limetta* essential oil against different types of radicals. The bactericidal properties of the essential oil were also significant; however, they were less than that of the D-Limonene and gentamicin. Likewise, the essential oil is found to inhibit the biofilm formation properties of different bacteria. The cytotoxic effect of the *C. limetta* essential oil was noticed against breast cancer cells of different receptors, specificity. Furthermore, the mechanism of action is found to be mediated through reactive oxygen species-mediated mitochondrial toxicity. Hence, the essential oil from the peel wastes of *Citrus limetta* is found to be pharmacologically active and emerges as a potential antibacterial and cytotoxic agent.

## Figures and Tables

**Table 1:** Chemical constituents of the peel of the *C. limetta* essential oil.

Peak No.	Retention Time	Component	Retention Index		%Relative Area ^a^
			Calculated	Library	
1	5.56	α-pinene	939	938	1.17 ± 0.96
2	6.41	α-myrcene	985	983	4.85 ± 0.67
3	6.76	3-carene	1011	1012	1.20 ± 0.08
4	7.09	D-limonene	1029	1029	85.71 ± 0.41
5	8.12	Linalyl acetate	1237	1133	1.64 ± 0.37
6	8.97	Citronellal	1153	1141	0.36 ± 0.06
7	9.48	Terpinen-4-ol	1175	1176	0.36 ± 0.28
8	9.70	α-Terpineol	1178	1180	0.44 ± 0.03
9	10.31	cis-p-mentha-1(7),8-dien-2-ol	1190	1195	0.43 ± 0.43
10	15.82	Guaia-1(10),11-diene	1490	1488	0.32 ± 0.91

^a^ Relative area (peak area relative to the total peak area).

**Table 2:** Radical quenching abilities of essential oil from *Citrus limetta* essential oil (CLEO) are expressed in terms of IC_50_ (µg/mL).

	DPPH Radical Scavenging	ABTS Radical Scavenging	H_2_O_2_ Radical Scavenging	Ferric Reducing Antioxidant Power	Lipid Peroxidation Inhibition
CLEO	11.35 ± 0.51 *	10.36 ± 0.55 *	8.28 ± 0.35 *	8.67 ± 0.21	30.19 ± 0.27 *
D-limonene	48.49 ± 0.22	41.22 ± 0.13	20.67 ± 0.34	19.08 ± 0.33	58.16 ± 0.43
Ascorbic acid	9.57 ± 0.75 *	11.08 ± 2.11 *	19.62 ± 1.60	3.41 ± 0.29 *	65.98 ± 1.95

(* *p* < 0.05).

**Table 3:** Anticancer activity expressed in terms of IC_50_ (µg/mL) value of the *Citrus limetta* peel essential oil.

	MCF-7	MDAMB231
CLEO	47.31 ± 3.11	55.11 ± 4.62
D-Limonene	392.57 ± 5.29	428.33 ± 4.61
Cyclophosphamide	10.02 ± 0.38	9.37 ± 0.25

**Table 4:** Bactericidal efficacy of the *Citrus limetta* (CLEO) essential oil, D-Limonene, and gentamicin (GM) by disc diffusion assay in MHA plates.

Strain	Zone of Inhibition (mm)		
	CLEO	D-Limonene	GM
*Escherichia coli*	13.5 ± 0.4	16.7 ± 0.2	22.5 ± 0.1
*Pseudomonas aeruginosa*	16.8 ± 0.2	18.7 ± 0.2	19.5 ± 0.2
*Staphylococcus aureus*	17.1 ± 0.5	20.9 ± 0.3	23.0 ± 0.1
*Salmonella enterica*	15.9 ± 0.3	17.1 ± 0.4	19.5 ± 0.3

**Table 5:** Minimum inhibitory concentrations (mg/mL) of the *Citrus limetta* (CLEO) essential oil and standard gentamicin (GM) against selected microbial strains.

Bacteria	MIC Concentration (mg/mL)		
	CLEO	D-Limonene	GM
*Escherichia coli*	0.50 ± 0.03 *	0.0625 ± 0.02	0.0312 ± 0.01
*Pseudomonas aeruginosa*	0.75 ± 0.03	0.0312 ± 0.01	0.0312 ± 0.01
*Staphylococcus aureus*	0.50 ± 0.02	1.25 ± 0.1	1.5 ± 0.3
*Salmonella enterica*	0.625 ± 0.03 *	0.0312 ± 0.00	0.0312 ± 0.01

(*p < 0.05).

**Table 6:** The percentage inhibition of antibiofilm formation activity of the *Citrus limetta* (CLEO) essential oil and standard gentamicin (GM) against selected microbial strains (at 0.5 mg/mL).

	Percentage Inhibition		
	CLEO	D-Limonene	GM
*Escherichia coli*	90.6 ± 1.6	100	100
*Pseudomonas aeruginosa*	92.19 ± 1.2	100	100
*Staphylococcus aureus*	95.6 ± 2.1	100	100
*Salmonella enterica*	93.8 ± 1.5	100	100

## Data Availability

Data may be made available upon valid request.

## Supplementary Materials

The following supporting information can be downloaded at: https://www.mdpi.com/article/10.3390/molecules27238329/s1, Figure S1. The untreated MCF7 cells (a) and the cytotoxicity of the *Citrus limetta* essential oil (b), D-Limonene (c), and cyclophosphamide (d); Figure S2. The untreated MDA-MB-231 cells (a) and the cytotoxicity of the *Citrus limetta* essential oil (b), D-Limonene (c), and cyclophosphamide (d).

## Sample Availability Statement

Samples of the compounds are available from the authors.
