# Supplementary material for: Chemical Composition, Antioxidant, Anti-Bacterial, and Anti-Cancer Activities of Essential Oils Extracted from Citrus limetta Risso Peel Waste Remains after Commercial Use
Source: Molecules. 2022 Nov 29;27(23):8329. doi: 10.3390/molecules27238329 (PMC9735939; doi:10.3390/molecules27238329)
Supplement: Supplementary file 1 [file molecules-27-08329-s001.zip › molecules-2011563-supplementary.pdf]

Supplementary Materials

Chemical Composition, Antioxidant, Anti-Bacterial, and Anti-Cancer Activities of Essential Oils Extracted from *Citrus limetta* Risso Peel Waste Remains after Commercial Use

Arunaksharan Narayanankutty [1,*], Naduvilthara U. Visakh [2], Anju Sasidharan [1], Berin Pathrose [2], Opeyemi Joshua Olatunji [3,4,*], Abdullah Al-Ansari [5], Ahmed Alfarhan [5] and Varsha Ramesh [6]

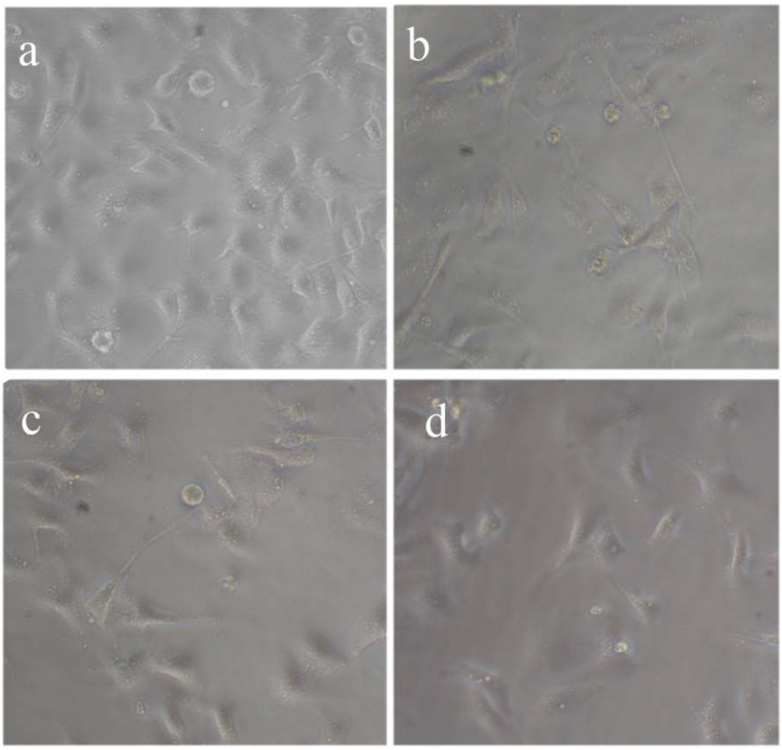

Figure S1. The untreated on MCF7 cells (**a**) and the cytotoxicity of *Citrus limetta* essential oil (**b**), D-Limonene (**c**) and cyclophosphamide (**d**).

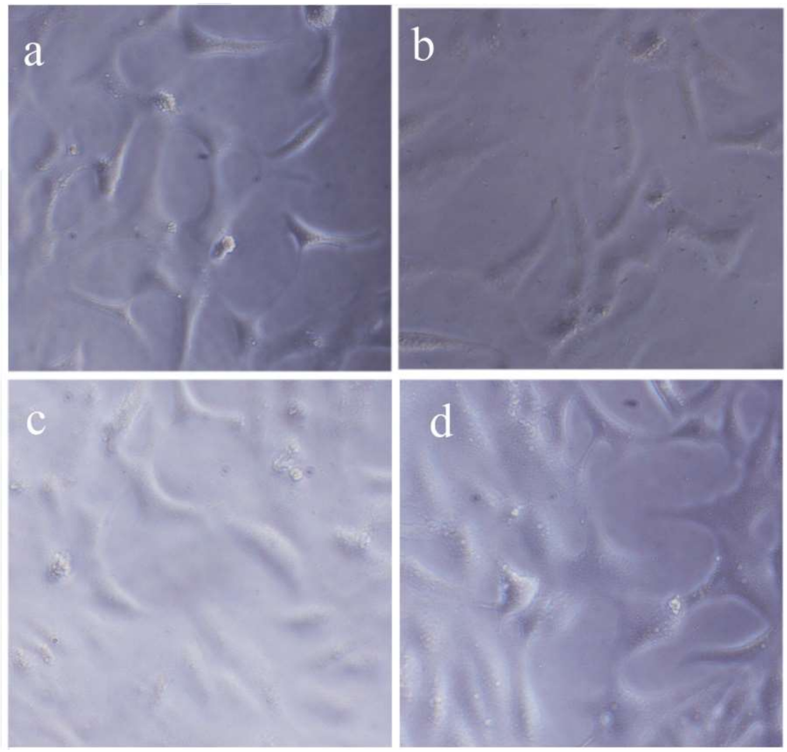

Figure S2. The untreated on MDA-MB-231 cells (**a**) and the cytotoxicity of *Citrus limetta* essential oil (**b**), D-Limonene (**c**) and cyclophosphamide (**d**).
